# Esophagectomy for very low body weight: a case report and literature review

**DOI:** 10.3389/fonc.2025.1524764

**Published:** 2025-06-23

**Authors:** Tianyu Zhang, Ruyuan He, Yongguang Xiao

**Affiliations:** Department of Thoracic Surgery, Renmin Hospital of Wuhan University, Wuhan, China

**Keywords:** esophageal cancer, malnutrition, low body weight, neoadjuvant therapy, postoperative care

## Abstract

Esophageal cancer (EC) has been widely concerned because of its high incidence, high mortality and high recurrence rate. However, the traditional surgical treatment of EC has poor prognosis and high recurrence rate. Malnutrition is the most common problem in patients with EC before and after surgery, and it has gradually attracted people’s attention because of its important impact on the efficacy, prognosis and treatment of patients with EC. However, EC patients are often accompanied by malnutrition, and their low BMI is often one of the difficulties in the treatment. Here, we report a very low body weight patient with advanced EC treated with neoadjuvant chemoradiotherapy combined with surgery. Our case shows that very low body weight caused by malnutrition should not be a contraindication for surgical treatment, and combined treatment for EC remains to be popularized.

## Introduction

Esophageal cancer (EC) is one of the most common malignant tumors of the digestive tract (1). Due to its characteristics of high prevalence, high mortality, late diagnosis and poor prognosis, it has been an urgent problem to find a better treatment for EC (2). Surgery is the preferred radical treatment for patients with EC, but the problems of high recurrence rate, poor prognosis and low survival rate still need to be solved urgently (3). With the rise of neoadjuvant therapy, the multidisciplinary treatment of EC has also developed. Malnutrition is a common and serious problem before and after surgery for EC (4, 5). Low body weight and low BMI caused by malnutrition seem to be related to postoperative recurrence rate, mortality and overall survival (6–8). Besides, malnutrition also affects the choice and efficacy of treatment (9). Here, we report a very low body weight patient with advanced EC who underwent neoadjuvant therapy followed by surgical resection, in order to provide a new perspective for the clinical treatment of EC.

## Case report

The patient was a 65-year-old female with a height of 150cm and weight of 29 kg, and a body mass index(BMI) of only 12.9. She was admitted to the hospital due to eating obstruction and vomiting for more than 2 months, which was aggravated for 1 consecutive day. Two months ago, she manifested difficulty in ingesting food without any evident inducement, and was occasionally accompanied by vomiting. The condition progressively aggravated. Subsequently, the patient visited a local hospital. The upper gastrointestinal barium meal indicated a malignant neoplastic lesion in the thoracic segment of the esophagus, and the carcinoembryonic antigen(CEA) was 5.16 ng/ml. The local hospital diagnosed erosive gastritis, gastric ulcer (H1), and esophageal cancer. Gastric protection and nutritional treatment were administered to the patient, but the patient’s condition did not show significant improvement. Three weeks ago, the patient began to have obstructive ingestion without any obvious cause. One day ago, the condition worsened, and the patient was unable to take in food, thus coming to our hospital for medical consultation. Outpatient gastroscopy revealed erosive gastritis, gastric ulcer, and the distance from the tumor to the incisor tooth was 25-30cm. Pathology suggested squamous cell carcinoma. After Multi-Disciplinary Treatment(MDT) consultation in the outpatient department, neoadjuvant therapy was first recommended and she was admitted to the oncology department of our hospital. She had been suffering from cachexia and had a history of gastric ulcer, chronic gastritis, and chronic bronchitis for many years. Upon inquiry, the patient continued to have a low BMI for many years, and her BMI index was maintained at about 13 for many years.

**Figure 1:**
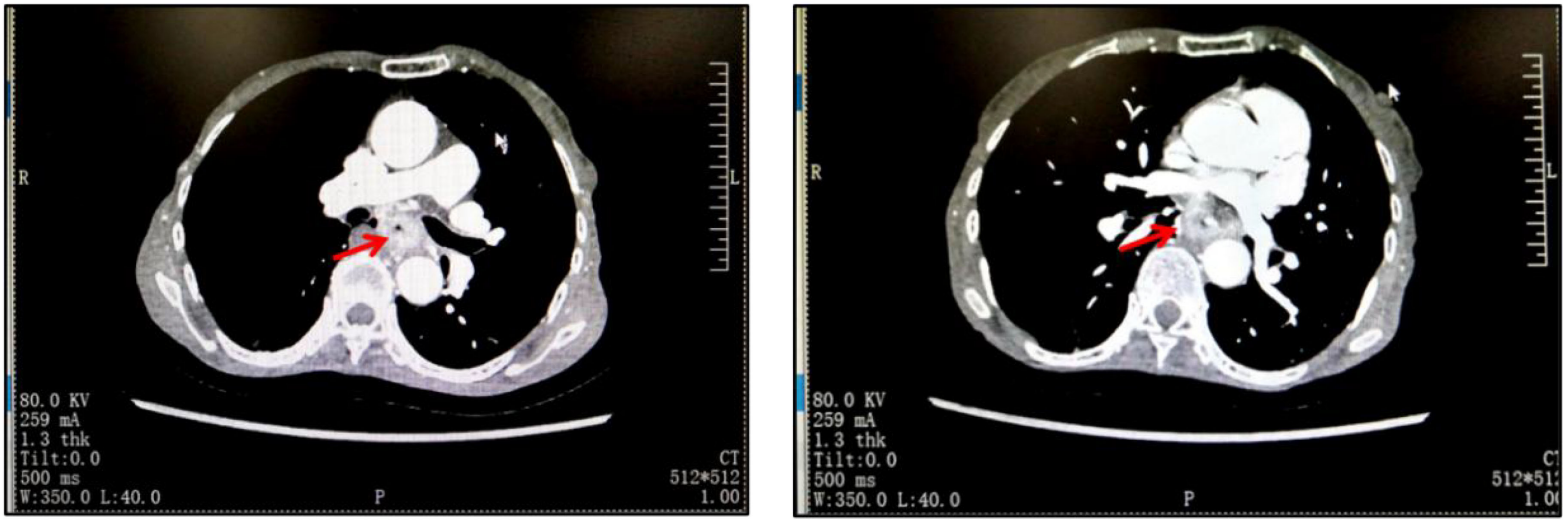
The imaging results of the patient on admission showed that the wall of the middle esophagus was thickened.

The admission physical examination showed that the CEA was 19.51 ng/mL, red blood cells were 3.57*10^12^/L, hemoglobin was 104 g/L, and there was mild anemia. Prealbumin and albumin were decreased to a relatively low level, respectively 153.11 mg/L and 35.5 g/L. Chest CT showed thickening of the middle wall of the esophagus (**Figure 1**). Endoscopic ultrasound suggested a protrusion at 30 cm from the incisors of the esophagus with erosive foci on the surface. PET-CT showed: 1. The middle thoracic esophagus was enlarged with increased metabolism, and mediastinal and hilar lymph nodes were enlarged, with some showing increased metabolism. All of these considerations malignant lesions. 2. Small nodules near the interlobar fissure of the right lung, with low metabolism, were considered inflammatory proliferative lesions; right paraglottic pleural thickening; 3. No other signs of metastasis. The biopsy of the endoscopic ultrasound showed squamous cell carcinoma, and the biopsy of the esophagus showed a new growth at 25-30 cm from the incisors, with the tumor infiltrating the entire layer of the esophagus. The pathological report suggested that the esophageal biopsy result was *in situ* squamous cell carcinoma, as the tissue sampling was superficial, the invasion was not excluded; the biopsy of the antrum of the stomach showed mild chronic inflammation and mild activity of the mucosa. The patient had lymph node metastasis in group 7, and the other lymph nodes were negative. Based on the preoperative examination materials, the final diagnosis for this patient with a low BMI was esophageal middle segment squamous cell carcinoma (cT3N1M0), cachexia, gastric ulcer, chronic gastritis, chronic bronchitis, anemia and hypoproteinemia. In addition, preoperative examination indicated that she had cardiovascular diseases such as coronary heart disease, tentorium arachnoid cyst of the cerebellum, bilateral maxillary sinuses and ethmoid sinusitis, etc., considering her basic physical fitness level, we rejected the treatment with immune checkpoint inhibitors (ICIs).

**Figure 2:**
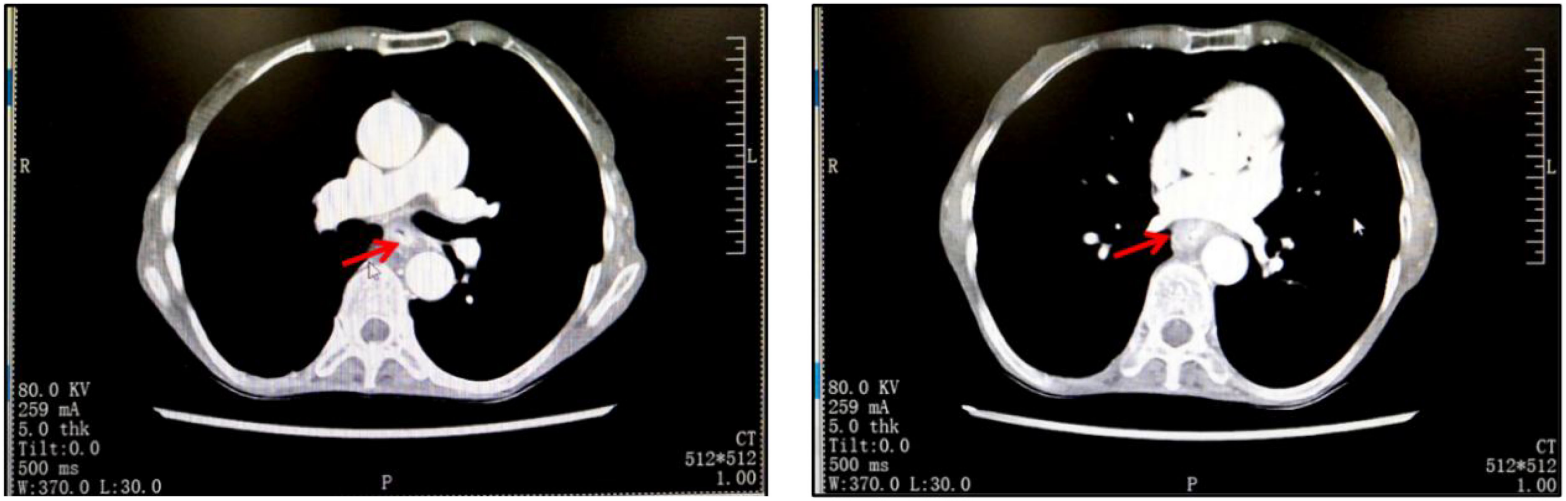
Imaging changes of the patient approximately 2 months after neoadjuvant therapy.

Due to the fact that although the patient was in cachexia and had a low BMI for a long time, surprisingly, her nitrogen balance was still within the normal range. Therefore, we did not overly focus on dealing with her malnutrition problem, but after completing the basic examination and nutritional assessment, we formulated a targeted treatment plan and performed neoadjuvant treatment for her. The first course of chemotherapy was albumin-bound paclitaxel and cisplatin, followed by four weeks of mediastinal CTV1 40Gy//20F radiotherapy, and the patient could not tolerate 20Gy. The second course of chemotherapy was performed in the following four weeks. After neoadjuvant therapy, chest CT showed that the lesion was significantly reduced (**Figure 2**). Four weeks later, the patient underwent a three-incision thoracoscopic and laparoscopic esophagectomy (McKeown’s procedure) at the thoracic surgery department of our hospital.The patient was diagnosed with esophageal squamous cell carcinoma (T4N1M0) after surgery. The postoperative pathological examination showed that the specimen of esophageal mass could be seen in the mucosa and submucosal tissue layer under histomicroscopy with ulcer formation, infiltration of a large number of neutrophils and lymphocytes, focal histiocyte and multinucleated giant cell reaction, and a small number of regressed squamous cell carcinoma cells could be seen in the superficial myofibroblast layer, which was consistent with the changes of esophageal cancer after radiotherapy, and no cancer could be seen in the rest of the esophageal remnants, gastric remnants, and the neighboring lymph nodes (beside the left laryngeal reentrant nerve, right laryngeal reentrant nerve and beside pancreatic nerve). The rest of the esophageal stump and neighboring lymph nodes (left paraglottic nerve, right paraglottic nerve, and paracardia) were examined without cancer. After surgery, she had a consultation with the nutrition department to formulate a nutrition plan, and the nutrition department thought that she was in total parenteral nutrition, with an energy supply of 1140kcal/day, which was sufficient, and started pumping saline through the jejunum tube from the same day of the consultation, and if there was no discomfort, she could be given enteral nutrition suspension Pepcid, with a slow pumping rate of 200 ml/day at the beginning. Wound recovery was assessed by means of iodography or methylene blue on postoperative day 7, and if recovery occurred, a fluid diet was initiated. Nutritional supplements were calculated on the basis of the patient’s body weight, and unconsumed food (calculated food intake minus the patient’s actual food intake) was administered through a jejunal feeding tube. The patient was started on a liquid diet one week after surgery and was discharged on the 9th postoperative day. One month after surgery, she was unable to tolerate chemotherapy at our hospital. According to the 2021 CSCOEC guidelines, for patients who underwent R0 resection and did not achieve pCR after neoadjuvant chemotherapy, adjuvant immunotherapy (nivolumab) can be performed. Therefore, she received immunotherapy monotherapy. Currently, she still comes to our hospital for subsequent adjuvant treatment regularly, with her BMI remaining at around 13, mainly on a liquid diet.

## Discussion

In recent years, certain progress has been made in the treatment of esophageal cancer. In addition to common surgical treatments, neoadjuvant therapy for EC has gradually become an important direction of exploration in clinical treatment. Neoadjuvant therapy includes neoadjuvant radiotherapy alone, neoadjuvant chemotherapy alone, and neoadjuvant concurrent chemoradiotherapy (NCRT) (10). Neoadjuvant concurrent chemoradiotherapy combined with surgery is currently the best treatment for locally advanced esophageal squamous cell carcinoma (11), which improves the R0 resection rate, Disease-Free Survival (DFS) and overall survival (OS) with acceptable safety (12–15). Based on the characteristics of high somatic mutation rate and strong immunogenicity of EC, the 2021CSCO esophageal cancer guidelines have clearly indicated that preoperative neoadjuvant therapy is recommended for resectable EC in stage T1b-4a/N. The recommended timing of surgery after neoadjuvant therapy is 4-8 weeks after the end of radiotherapy and chemotherapy if the patient’s physical condition is acceptable, and 3-6 weeks after the end of chemotherapy. The advantage of neoadjuvant therapy is to reduce tumor stage and increase R0 resection rate; Inhibition of tumor cell activity and reduction of intraoperative dissemination (16, 17); The patient’s tolerance is better than that of postoperative adjuvant therapy. The effectiveness of preoperative treatment can be objectively evaluated from the pathological indicators.

Most patients with esophageal cancer lose a large amount of weight before diagnosis (5), usually manifested as a low BMI (<18.5kg/m2), because the tumor can cause esophageal obstruction, causing dysphagia and resulting in reduced food intake. But patients with extremely low BMI (<13kg/m^2^) are generally rare and only occur in two situations: either dysphagia caused by esophageal cancer lasts for a relatively long period, and that mean the patient is in a negative nitrogen balance state for a long time, resulting in sustained energy consumption and the formation of extremely low BMI (18); or the patient is already in an extremely low BMI state before the onset of the esophageal cancer, and the body has adapted to low energy intake even if dysphagia occurs, and the reduced food intake will not greatly affect weight loss. But such patients have often-concurrent anemia and hypoalbuminemia, and for example, our patient’s hemoglobin level is only 104. So before surgery these patients not only need to correct negative nitrogen balance, but also need to correct hypoalbuminemia and anemia. However, because our patient had been plagued by digestive diseases and had a low BMI for a long time, her preoperative examination showed that her nitrogen balance was still within the normal range despite low albumin and prealbumin, although the patient had eating and swallowing obstruction, it was limited to hard food or a large number of soft foods. There was no obvious eating obstruction for liquid diet, and after the obstruction appeared, the patient did not have significant further weight loss or malnutrition such as hypoproteinemia and anemia. Therefore, we did not take special treatment measures to correct the nutritional status of the patient during the preoperative period, but instead focused more on the combination therapy, while regularly assessing her nutritional status by means of blood measurements and albumin-nitrogen balance evaluation, etc., as evaluation criteria of nutritional status.

The treatment of esophageal cancer in patients with very low BMI (body mass index) faces many challenges, which are mainly related to the patient’s weight status, nutritional status, and limitations in physical functioning. Patients with very low BMI are usually associated with severe malnutrition, resulting in decreased immunity and increased risk of infection. Studies (19) have shown that BMI is associated with good long-term survival of EC patients receiving different therapies in China. Elderly patients in the low BMI group are more likely to have anemia and hypoproteinemia before operation, and are more likely to have lung infection and incision infection than those in the normal BMI group. During NRCT, the dose of radiotherapy and the toxicity of chemotherapeutic agents are usually related to the patient’s weight. Malnutrition can also affect patients’ tolerance to surgery and radiotherapy and chemotherapy, and postoperative radiotherapy and chemotherapy are often difficult to implement as planned. Previous experience has shown that most patients have low PS scores due to individual differences in the process of dietary rehabilitation after EC surgery. The preoperative PS score of our patient was 0, which was one of the reasons for her successful neoadjuvant therapy.

In fact, the association between body mass index and esophageal cancer patients has been reported in the past (20, 21), but there have been few case reports of esophageal cancer patients with low body weight. Most studies have emphasized that patients with low BMI should be given priority for enteral or parenteral nutrition support before surgery due to their high risk of malnutrition to improve surgical tolerance (22). In addition, some studies have pointed out that patients with low BMI may be more inclined to minimally invasive surgery (such as thoracoscopic esophagectomy) due to the high risk of postoperative complications, but cardiopulmonary function should be strictly evaluated (23). It has also been suggested that patients with low BMI may reduce the scope of surgery or delay surgery or palliative care due to more comorbidities (such as diabetes and cardiovascular disease).

In this patient’s case, despite cachexia and a personal history of digestive diseases for many years, her nitrogen balance remained normal, indicating that her low BMI was not caused by reduced food intake due to obstructed eating due to esophageal cancer. Her preoperative albumin, hemoglobin, and other key indicators remained within normal ranges. Her nitrogen balance was still normal, and therefore, in this case, we did not think that aggressive correction of malnutrition was necessary, which is what made this patient special. The low BMI is not a contraindication for surgery. According to the 2024CSCO guidelines for esophageal cancer, artificial nutrition is recommended for patients with malnutrition (weight loss within 6 months) >10%, BMI<18.5kg/m^2^, or serum albumin <30g/L in patients without liver dysfunction. In fact, based on the most recent 2024 guideline, we also see that this patient does not fall within that recommendation. We believe that low BMI and advanced age with multiple underlying diseases should not be contraindicated to surgery. In addition, low body weight can affect NCRT in cancer treatment, and fortunately, in the absence of strong nutritional intervention, she was able to complete NCRT on the basis of her previous weight baseline, although our initial intention was to stop and consider alternative therapies if she became medically intolerable.

In conclusion, our case shows that low BMI does not necessarily reduce the postoperative survival of ESCC patients, so patients with low BMI can be safely treated with surgery, but intraoperative prevention and postoperative monitoring should be strengthened. In addition, we emphasize that neoadjuvant immunotherapy is worthy of promotion as postoperative adjuvant therapy, but it is worth noting that the optimal treatment regimen of neoadjuvant concurrent chemoradiotherapy remains to be explored, including but not limited to the optimal radiation dose, chemotherapy regimen, chemotherapy cycle, whether it can be performed sequentially, and the interval between surgery. Further improvement in the assessment of response to neoadjuvant therapy should also be the focus of future research.

## Data Availability

The original contributions presented in the study are included in the article/supplementary material. Further inquiries can be directed to the corresponding authors.
